# Mechanical Properties and Microstructure of Decellularized Brown Seaweed Scaffold for Tissue Engineering

**DOI:** 10.3390/bioengineering12090943

**Published:** 2025-08-31

**Authors:** Svava Kristinsdottir, Ottar Rolfsson, Olafur Eysteinn Sigurjonsson, Sigurður Brynjolfsson, Sigrun Nanna Karlsdottir

**Affiliations:** 1 Industrial Engineering, Mechanical Engineering and Computer Science Department, University of Iceland, 102 Reykjavik, Iceland; sb@hi.is (S.B.); snk@hi.is (S.N.K.); 2 School of Health Sciences, Medical Department, University of Iceland, 102 Reykjavik, Iceland; ottarr@hi.is; 3 School of Science and Engineering, Reykjavik University, 102 Reykjavik, Iceland; oes@ru.is

**Keywords:** decellularization, seaweed, biomaterials, extracellular matrix, scaffold, mechanical properties, porosity and microstructure

## Abstract

In response to the growing demand for sustainable biomaterials in tissue engineering, we investigated the potential of structurally intact brown seaweed scaffolds derived from *Laminaria digitata* (*L.D*.) and *Laminaria saccharina* (*L.S*.), produced by a detergent-free, visible-light decellularization process aimed at preserving structural integrity. Blades were submerged in cold flow-through and aerated water with red (620 nm) and blue (470 nm) light exposure for 4 weeks. Histology, scanning electron microscopy (SEM), and micro-computed tomography (micro-CT) analyses demonstrated that the light decellularization process removed cells/debris, maintained essential structural features, and significantly increased scaffold porosity. Mechanical property analysis through tensile testing revealed a substantial increase in tensile strength post decellularization, with *L.D.* scaffolds increasing from 3.4 MPa to 8.7 MPa and *L.S.* scaffolds from 2.1 MPa to 6.6 MPa. Chemical analysis indicated notable alterations in polysaccharide and protein composition following decellularization. Additionally, scaffolds retained high swelling and fluid absorption capacities, critical for biomedical uses. These findings underscore that the decellularized *L.D.* and *L.S.* scaffolds preserved structural integrity and exhibited enhanced mechanical properties, interconnected porous structures, and significant liquid retention capabilities, establishing them as promising biomaterial candidates for soft-tissue reinforcement, wound care, and broader applications in regenerative medicine.

## 1. Introduction

Scaffolds are central to tissue engineering (TE) and regenerative medicine (RM) because of the three-dimensional (3D) environment that facilitates cellular infiltration, adhesion, and tissue regeneration [1]. Scaffolds are typically categorized based on their method of production, such as those synthesized through 3D printing, electrospinning, or other techniques vs. those generated by decellularizing living tissues to retain the natural structure of the extracellular matrices [2]. The synthesis of scaffolds allows for precise control over their composition, enabling the integration of antibacterial agents, adjustment of pore sizes to meet specific structural requirements prior to implantation, and even incorporation of selected cells into the matrix using 3D printing technology [3,4,5]. The use of the extracellular matrix (ECM) from living tissues provides the natural microstructure suitable for the receding or repopulation of new cells and distribution of nutrition within the structure. It is, however, essential to remove the original cells from the tissue via a decellularization process to minimize antigen load [6]. Decellularized tissue requires physical, chemical, or enzyme process or a combination of all to remove cellular antigens while attempting to retain the native structure and bioactivity [7,8]. Chemical decellularization commonly uses detergents, sometimes including enzymes, to burst cells and remove immunogenic cellular material, but such treatments can disrupt the ECM composition and structure and, if residual reagents are not thoroughly removed, impair cytocompatibility and cell proliferation [9,10,11]. The clinical application of the ECM in advanced wound healing and reconstructive procedures, including hernia and breast reconstruction surgeries, frequently utilizes collagen sourced from animal tissues [12,13,14]. Although effective, these materials can be expensive, carry immunological risks, and raise cultural or religious concerns, motivating the exploration of non-animal alternatives [15,16]. Plant- and algae-derived scaffolds, whose frameworks are rich in cellulose, offer a biocompatible and mechanically tunable option [17]. Over the last decade, “cross-kingdom” scaffolds from decellularized plants (e.g., apple hypanthium, spinach leaf vasculature) have supported viable mammalian cell culture and, in some cases, angiogenesis in vivo, highlighting the feasibility of cellulose-based matrices for TE [18,19,20]. Marine macroalgae (seaweed) are largely free of lignin compared to terrestrial plants yet retain cellulose microfibrils that provide rigidity [21,22,23]. In addition to cellulose, macroalgae contain polysaccharides such as alginate, laminarin, and fucoidan, each widely explored for biomedical applications [24,25,26]. A further advantage of marine algae is the rapid biomass accumulation; the low lignin content simplifies cellulose purification and reduces the need for harsh chemicals [27]. In brown seaweed (Phaeophyceae), cell walls are comprised of cellulose microfibrils reinforced by alginate and fucose that contain sulfated polysaccharides (fucoidans) while laminarin is the major storage glucan [28,29]. These components modulate the mechanical and hydration with potential independent biomedical properties [30,31]. Morphologically, the laminaria kelp (e.g., *Laminaria digitata* (*L.D.*) and *Saccharina latissima* (*Laminaria saccharina* (*L.S.*)) exhibit a stratified thallus with an outer epidermis/meristoderm, cortical layer, and central medulla containing conducting trumpet hyphae [32,33]. This hierarchical microstructure, reinforced by cellulose and alginate, may offer a natural framework for cellular attachment, nutrient transport, and mechanical stability. Indeed, a hierarchical microstructure has repeatedly been shown to be important for TE and RM as it features structures and pores of nano, micro, and macro scales mimicking the natural structure of living tissues [34,35]. Recent studies on hieratically produced nanofiber scaffolds for periodontitis-related alveolar bone defects have reported reduced inflammatory response and promoted alveolar bone regeneration [36]. Macroalgal polysaccharide extracts have already been established within TE/RM. Alginate is widely used in advanced wound care and as a base for bioink for 3D printing due to its biocompatibility and mild gelation [37]. Recent reviews have summarized clinical use for alginate-base products for exudative and infective wounds as well as peptide-modified alginates that promote cell vs. material interaction [38]. Additionally, laminarin is being explored as a bioactive component in biomedical composites and fucoidan shows immunomodulatory and pro-healing effects in hydrogel systems [30]. However, the biomedical application of structurally intact brown seaweed extracellular matrix (ECM), which preserves its native architecture as opposed to utilizing reconstituted or isolated polysaccharides, has only recently begun to emerge. Green-algal cellulose scaffolds from *Ulva* and *Cladophora* support fibroblast growth in vitro and angiogenesis in vivo, demonstrating the baseline biocompatibility of seaweed-derived ECM [39]. In recent studies, decellularized green and brown macroalgae (*Ecklonia radiata*) have been converted into cellulose matrices and systematically analyzed, highlighting both the potential of seaweed-derived extracellular matrix materials and the necessity to optimize decellularization methods while maintaining the structural integrity of the scaffolds [10]. Decellularization is a critical step for producing an ECM with minimal immunogenicity while preserving native architecture and mechanics. The chemical decellularization of polysaccharide-rich algae, in which ionic interactions such as alginate cation cross-linking significantly influence cell wall mechanics, may compromise the structural integrity of the ECM while remaining ineffective at complete cellular removal from the ECM [10,11]. This study examines two brown seaweed species of significant ecological and economic value, *L.D.* and *L.S.*, which are abundant in the North Atlantic—particularly along the Icelandic coast—and are also extensively cultivated throughout Europe [40,41]. Tensile strength assessments of native *L.D.* and *L.S.* (3.1–4.7 MPa) [23] suggest their potential suitability for soft tissue applications, displaying properties comparable to those of previously investigated biological scaffolds [1,2]. However, research on the intact, decellularized ECM derived from these species remains limited. A mild, non-chemical, light-assisted decellularization protocol facilitating the structure integrity of the seaweed scaffold was developed and tested on two species of brown seaweed, *L.D.* and *L.S*. Thawed blades of the seaweeds were mounted on frames and submerged in continuously refreshed, cold, turbulent water for four weeks. During immersion, blades were exposed to visible-spectrum light: red (≈620 nm) and blue (≈470 nm). Nine illumination sequences were screened; the sequence that achieved the most consistent nuclear and cell-debris clearance in both species was selected for subsequent scaffold production and testing. Decellularization was confirmed using hematoxylin and eosin (H&E) staining, with the quantitative evaluation of nuclear and cellular debris removal performed across specified blade regions by light microscopy. In this study, the criteria for successful decellularization included the absence of visible nucleated cells and effective clearance of cellular debris as assessed histologically by H&E staining. The resulting acellular matrices were characterized by chemical composition, microstructure, fluid absorption, porosity, and hydrated mechanical properties.

This study addressed the existing gap in decellularization methodologies by presenting an approach that yields structurally preserved macro- and microstructured ECMs from *L.D.* and *L.S*. It introduced a species-specific, non-chemical processing technique purposefully developed to retain architectural integrity for subsequent tissue engineering and regenerative medicine applications. This work contributes to the development of a sustainable, animal-free scaffold platform.

## 2. Materials and Methods

### 2.1. Sample Collection

Seaweed samples *L.D.* and *L.S.* were obtained from Eyjafjörður near Hauganes in the autumn of 2022 (GPS: coordinates 65.933373, −18.265253). Additional seaweed samples *L.D.* and *L.S.* were harvested along the Reykjanes Peninsula coastline, Iceland, during autumn of 2023 (GPS: 63.97056885259877, −22.75364300250477). All seaweed was rinsed with cool water, sorted manually, and frozen at −14 to −20 °C until further processing.

### 2.2. Decellularization

A novel method using visible light spectrum was developed and implemented to generate acellular ECM from the harvested *L.D.* and *L.S.* specimens. Briefly, this procedure involves mounting thawed seaweed on frames and submerged into plexiglass tanks with turbulent cool tab-water for a total duration of four weeks. Light exposure was confined to red light (λ = 620 nm) and blue light (λ = 470 nm) produced by Waveform Lighting, LLC, 4400 NE 77th Ave Ste 275 | Vancouver, WA 98662, USA. Ultraviolet (<400 nm) and infrared (>700 nm) wavelengths were excluded. Over the course of four weeks, nine distinct sequences of visible electromagnetic radiation exposures were systematically evaluated (Table 1).

**Figure 1:**
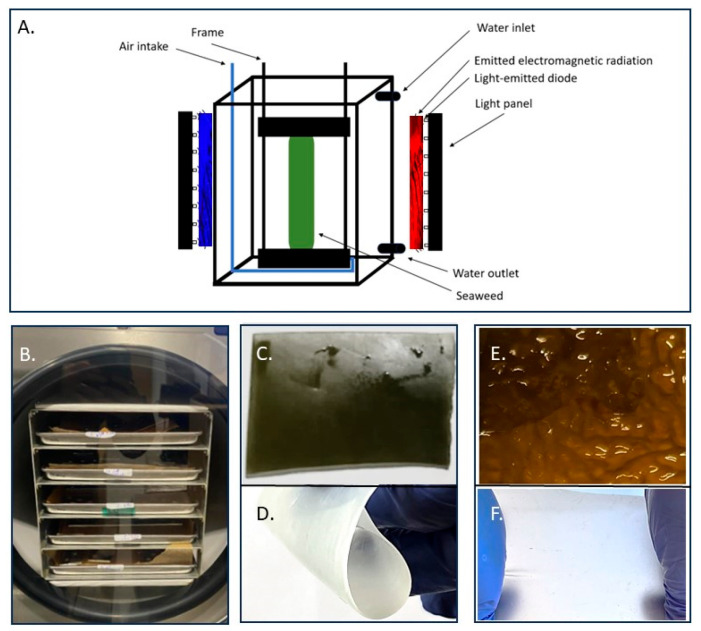
The decellularization unit developed and built at the University of Iceland. (**A**) Schematic of the decellularization-unit emitting electromagnetic radiation at wavelength 620 nm and 470 nm. (**B**) Samples lyophilized. (**C**) *L.D.* seaweed sample before decellularization. (**D**) Rehydrated acellular ECM *L.D*. (**E**) *L.S.* seaweed sample before decellularization. (**F**) Rehydrated *L.S.* acellular ECM.

Tanks operated with continuous inflow/outflow at 0.6–0.8 L·min^−1^ (mean water temperature ~6 °C), with bulk temperature maintained <12 °C by adjusting flow. Compressed air (1–2 psi; ~6.9–13.8 kPa) was introduced at the tank bottom to generate turbulent irritation on the surface of the seaweed (Figure 1A). The water temperature was maintained <12 °C by adjusting the waterflow into the tanks. Light panels were positioned on the opposite side of the tank emitting electromagnetic radiation at 620 nm and/or 470 nm, for total of 4 weeks. Finally, the samples were lyophilized at −50 °C and under a pressure of 0.5 pascal (Figure 1B).

### 2.3. Microstructural Analysis by Scanning Electron Microscopy

**Figure 2:**
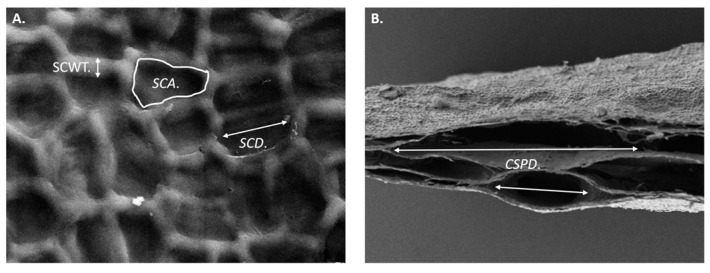
Microstructure measurements from SEM images explained. (**A**) The Surface Chamber Wall Thickness (SCWT), Surface Chamber Diameter (SCD), and Surface Chamber Area (SCA). (**B**) Cross-Sectional Pore Diameter (CSPD).

Both seaweed and scaffolds were lyophilized, sputter-coated with gold, and examined via scanning electron microscopy (FE-SEM, Zeiss Supra 25^®^, Oberkochen, Germany) for microstructural analysis. Images were acquired at accelerating voltages of 5 kV and 15 kV, with magnifications from 50× to 3000×. Subsequent additional measurements were conducted within the image analysis software ImageJ version 1.54i. As illustrated in Figure 2, four microstructural features were measured including the Surface Chamber Diameter (SCD) and Surface Chamber Area (SCA), a total of 50 measurements on the smallest visible chamber. Cross-Sectional Pore Diameter (CSPD) was measured (Figure 2B) with a total of 20 measurements. For the scaffolds only, a total of 20 measurements were conducted of the Surface Chamber Wall Thickness (SCWT) as consistent measurements could not be produced for the seaweed.

### 2.4. Histology (H&E) for Assessment of Decellularization

Following decellularization, four scaffold specimens were collected and fixed in 10% formaldehyde. Seaweed that underwent 4 weeks in tank without light and fresh seaweed specimens were fixed in parallel as controls. Tissues were processed for routine histology, mounted on glass slides, and stained with hematoxylin and eosin (H&E) to assess decellularization in both *L.D.* and *L.S*. Residual cells and cellular debris were evaluated on H&E-stained sections at high power (20× objective). Each slides contained four samples; six non-overlapping sites (one high-power field per site) were blindly evaluated per sample by counting cells and cellular debris. Each site received a 0–4 score:

Score scale:

0: No cells or cell debris per field;

1: Some cell debris but less than 50, no cells with nucleus per field;

2: Obvious cell debris but no cells with nucleus, more than 50 cell debris per field;

3: Few cells with nucleus (less than 50) per field;

4: Many cells with nucleus (more than 50) per field.

For each light sequence method, the six site scores were averaged to obtain a mean residual-cellularity score $\left(\bar{S}\right)$, and a lower $\left(\bar{S}\right)$ indicated more complete decellularization. Percent cell-removal success was then computed:

(1) $$Cell\;removal\;success\;(\%)\;=\left((S_{max}-\;\bar{S}\right)/S_{max})\cdot 100$$

### 2.5. Chemical Composition

All sample preparation and analyses were conducted at the Chemistry and Applied Mechanics Department of the Research Institute of Sweden (RISE) in Borås. Approximately 10 g of dried macroalgae, milled into a fine powder under liquid nitrogen, was analyzed. Elemental composition (Ca, Mg, Na, K, I) was determined using Inductively Coupled Plasma–Optical Emission Spectrometry (ICP-OES). Total protein content was quantified via carbon, hydrogen, and nitrogen analysis (CHN), employing a nitrogen-to-protein conversion factor of 4.12, as described previously by Biancarosa et al. [21].

Polysaccharides were extracted from macroalgae powder (~1 g) sequentially using hot water (90 °C, 20 h) and subsequently acidified water (pH 1.47, HCl, 100 °C, 2 h). Aliquots from both water and acid extracts were hydrolyzed in 2 M trifluoroacetic acid (80 °C, 2 h), derivatized using bis(trimethylsilyl)-trifluoroacetamide (BSTFA) containing 1% trimethylchlorosilane (TMCS), and quantified by Gas Chromatography–Mass Spectrometry (GC/MS). Laminarin concentration was estimated by measuring glucose. Alginate content was quantified from the acidic extract after dialysis (1 kDa cutoff), lyophilization, and sulfuric acid hydrolysis, using a carbazole-based colorimetric assay for released uronic acids. Additional carbohydrates (e.g., galactose, mannitol, cellulose) were similarly quantified through appropriate extraction, derivatization, and colorimetric assays, providing comprehensive polysaccharide profiles.

### 2.6. Swelling Ratio

Lyophilized seaweed and scaffolds were measured in thickness before and after rehydration in 0.9% (*w*/*v*) NaCl solution for 2 h using digital micrometer gauges. *L.D.* specimens were taken from the central region of the blade prior to its natural bifurcation while *L.S.* specimens were harvested from the lateral region of the blade approximately 5 cm below the stem. The swelling ratio was calculated according to

(2) $$swelling\;ratio={((t}_{i}-t_{0})/t_{0})\cdot 100$$

where $t_{0}$ is the initial thickness and $t_{i}$ is the thickness after 2 h of rehydration.

### 2.7. Absorption

Four lyophilized scaffold samples (*L.D.* and *L.S.*) were weighed prior to soaking in 0.9% (*w*/*v*) NaCl solution, then re-weighed at 1 h intervals for 5 h and after 24 h. The absorption ratio was calculated according to

(3) $$\%\;absorption\;ratio={((w}_{i}-w_{0})/w_{0})\cdot 100$$

where $w_{0}$ is the initial dry weight and $w_{i}$ is the wet weight at each time point [14].

### 2.8. Porosity

Three lyophilized seaweed samples (*L.D.* and *L.S.*) and their corresponding scaffolds were weighed (w_0_), and their geometry measured to calculate volume (V_s_). Samples were then immersed in pure ethanol (density = 0.79 g/mL at 20 °C) for 5 min, followed by agitation on a rocking table for 10 min to facilitate removal of trapped air. Subsequently, the samples were reweighed (w_1_), and porosity was calculated according to the following:

(4) $$\%\;porosity\;=\;{((w}_{1}-w_{0})/{\rho V}_{s})\cdot 100$$

### 2.9. Micro-CT Analysis

Lyophilized samples were scanned in air using a micro-computed tomography (CT) device, a Phoenix Nanotom S (Waygate Technologies/Baker Hughes Digital Solutions GmbH, Wunstorf, Germany), with an acceleration voltage of 70 kV and a source current of 110 μA. No X-ray filter was used, at the research facility IceTec (https://www.taeknisetur.is/?lang=en). A 10.0-fold magnification yielded a voxel size of 5.00 μm. Projection images were collected at 0.33° increments, each with a 3.0 s exposure time. Three projection images were integrated per rotation step, with a single blank image acquired before scanning. Reconstruction was performed with datos-x software, including translational motion compensation for sample shift between 0° and 360°. Virtual volumes were generated, and 3D or cross-sectional visualizations were produced in VGStudio Max 3.3 (Volume Graphics, Heidelberg, Germany). One image per scaffold type was subsequently used to measure the diameter of the interconnecting tunnels close to the surface of the scaffolds.

### 2.10. Mechanical Testing

Tensile testing was performed with a mechanical tester, a UniVert system (Cellscale), to measure ultimate tensile strength (*σ_uts_*) and Young’s modulus (E). Scaffolds were soaked in water for 2 h at room temperature while the frozen seaweed samples were only thawed. Dog-bone specimens were cut according to the ASTM D638-14 standard [42] for evaluation of the tensile properties of plastic materials. Thickness was measured after rehydration and thawing; each sample was kept moist until testing. For the tensile testing, a displacement rate of 7 mm/min was used with a 20 kgf load cell. Samples that ruptured outside the gauge section of the specimen were excluded. Stress–strain curves were generated to calculate the ultimate tensile strength (*σ_uts_*) according to the following:

(5) $$\sigma_{uts}=\frac{F_{max}}{A_{0}}$$

where $F_{max}$ is the force at break (N) and $A_{0}$ is the initial cross-sectional area (mm^2^). The strain (ε) is defined according to the following:

(6) $$\varepsilon =\frac{\delta }{L}$$

where δ is the elongation (mm) and *L* is the initial length (mm). Young’s modulus or elastic modulus (E) is defined as

(7) $$E=\frac{\sigma }{\varepsilon }$$

where *σ* is the stress (MPa) and ε the strain (mm/mm). Young’s modulus was defined as the slope of the linear portion of the stress vs. strain curve as previously defined by Ní Annaidh et al. [43].

### 2.11. Statistical Analysis

For parameters that satisfied the assumption of homogeneity of variance, one-way ANOVA followed by Tukey’s HSD post hoc tests was conducted. These tests included comparisons of Surface Chamber Wall Thickness (SCWT), micro-CT–cross-sectional pore size, and porosity within the scaffold groups, as well as strain at failure across seaweed and scaffold groups.

For parameters violating assumptions of homogeneity of variance, i.e., CSPD, SCD, SCA, tensile strength, Young’s modulus, and swelling ratio, Welch’s ANOVA was used, followed by Games–Howell post hoc testing for pairwise comparisons across seaweed and scaffold groups. A repeated-measures ANOVA was conducted to test for differences in absorption ratio across time and scaffold groups. When the assumption of sphericity was violated, the Greenhouse–Geisser correction was applied. A linear mixed-effects model with post hoc pairwise comparisons was used to explore group differences at each time point.

Statistical analyses were performed in RStudio, and significance was set at *p* < 0.05 or less. Significance level notation as follows: * *p* < 0.05, ** *p* < 0.01, *** *p* < 0.001, **** *p* < 0.00001, and ns (not significant).

## 3. Results

### 3.1. Visible-Light Decellularization Produces Acellular Seaweed Scaffolds with Preserved Architecture and Selective Biomatrix Retention

**Figure 3:**
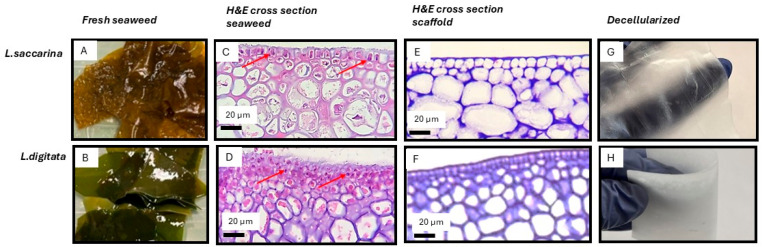
Macroscopic and histological comparison of seaweed and decellularized scaffolds. Macroscopic images and cross-sectional H&E micrographs of *L.S.* and *L.D.* seaweed with the corresponding decellularized scaffold are shown. The seaweed tissue contained numerous embedded cells whereas decellularized scaffolds showed no detectable cellular material by H&E staining, as well as showing preserved extracellular matrix (ECM) architecture. (**A**,**B**) Macroscopic images of the *L.S.* and *L.D.* seaweed. (**C**,**D**) H&E-stained fresh cross-sections of seaweed *L.S.* and *L.D.*, respectively; red arrows indicate representative cells. (**E**) H&E-stained cross-sections of decellularized *L.D.* scaffold showing an acellular ECM following red-light and blue-light sequence decellularization method. (**F**) H&E-stained cross-sections of decellularized *L.S.* scaffold showing an acellular ECM following red-light and combined blue + red-light sequence decellularization method. (**G**,**H**) Macroscopic images of decellularized rehydrated scaffolds (*L.S.* and *L.D.*, respectively) demonstrating structural integrity following decellularization.

Hematoxylin and eosin (H&E) staining revealed the seaweed and resulting scaffold in varying shades of purple while cells appeared with light pink cytoplasm and dark blue nuclei (Figure 3).

**Figure 4:**
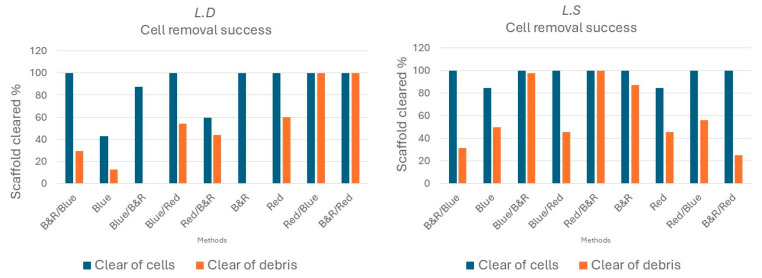
Decellularization of *L.D.* and *L.S.* seaweed under the 9 nine different light exposure sequences. For *L.D.*, cell and cellular-debris removal was maximized by red light (2 weeks) followed by blue light (2 weeks), or by combined blue + red light (2 weeks) followed by red light (2 weeks). For *L.S.*, the most effective sequence was red light (2 weeks) followed by combined blue + red light (2 weeks).

H&E staining revealed sequence-dependent differences in decellularization across nine light-treatment protocols in the two seaweed species (Figure 3 and Figure 4).

Across both seaweed species, the decellularized scaffolds preserved a microarchitecture broadly similar to the original seaweed tissue. However, decellularization efficacy was sequence- and species-dependent (Figure 4). For *L.D.* seaweed, the most effective sequences by H&E scoring were the red → blue-light sequence, and the combined blue-and-red → red-light sequence. Exposure to red light alone demonstrated moderate decellularization of the *L.D* seaweed. The least effective sequence for *L.D.* was the red → blue-and-red, and only blue-light sequence. In contrast, *L.S.* showed the opposite preference, with the best results produced by the red → blue-and- blue → blue-and-red sequence, whereas only the red light and only-blue-light sequences were least effective.

**Figure 5:**
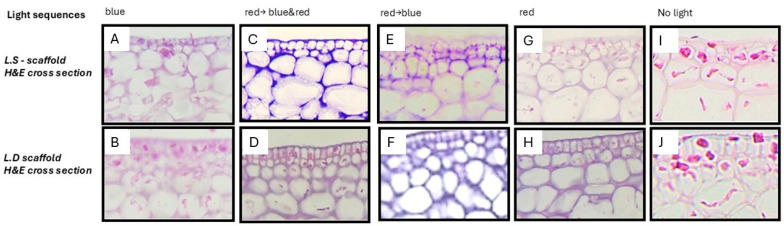
Histological examples of decellularization outcomes in *L.D.* and *L.S.* under visible-light regimens versus no-light control. H&E-stained cross-sections compare residual cells/cellular debris and ECM preservation after 4 weeks of treatment. In the no-light tank control, cells remain embedded within the ECM. (**A**,**B**) Blue light (4 weeks), residual cellular material persists; ECM architecture is preserved. (**C**,**D**) Red → blue and red (2 weeks each) *L.S.* appears largely acellular whereas *L.D.* shows some residual debris; ECM is preserved in both. (**E**,**F**) Red → blue (2 weeks each); *L.D.* appears well cleared and *L.S.* nearly cleared of cells; ECM is preserved. (**G**,**H**) Red light (4 weeks): both scaffolds appear nearly acellular; ECM is preserved. (**I**,**J**) No-light control: cells remain within the ECM. Note: R, red (620 nm); B, blue (470 nm); R + B, combined red + blue.

To facilitate cross-seaweed- species comparability and practical production, the red → blue illumination sequence (2 weeks each; 620 nm → 470 nm) was adopted for all subsequent scaffold preparations and testing. The resulting seaweed scaffolds lacked discernible peripheral cells while preserving the algal microstructure (Figure 5).

To assess if changes in cellular composition were accompanied by alterations to the chemical composition of the resulting scaffold, protein, carbohydrate, and mineral contents were measured and compared (Table 2 and Table 3). *L.S.* scaffolds showed a slight increase in total protein content relative to its original seaweed (5.5% vs. 3.6%) whereas the *L.D.* scaffold retained a comparable level (5.1% vs. 5.4%). Changes in carbohydrate composition between seaweed and scaffold samples were more pronounced than those observed in protein content, with changes varying according to the original seaweed species (Table 2). Galactose was represented in a low content in the *L.D.* seaweed (0.028 mg/g) but was increased by 14% in the scaffold. In contrast, the *L.S.* seaweed exhibited a higher galactose content (0.85 mg/g), which decreased by 90% in the scaffold. Mannitol content decreased substantially following decellularization, with reductions of approximately 64% in *L.D.* scaffolds (from 0.67 mg/g in seaweed to 0.24 mg/g) and 99% in *L.S.* scaffolds (from 25 mg/g to 0.25 mg/g), relative to their respective seaweeds. Cellulose content increased substantially in the *L.S.* scaffold, rising by approximately 69% (from 26% to 44% dry weight), whereas in the *L.D.* scaffold, it decreased slightly by about 9% (from 34% to 31% dry weight), relative to the seaweed. Both scaffolds exhibited reductions in alginate content, with a 25% decrease in the *L.D.* scaffold (from 85 mg/g in seaweed to 64 mg/g) and a 48% decrease in the *L.S.* scaffold (from 63 mg/g to 33 mg/g. The laminarin content was initially low in *L.D.* seaweed (<0.7 mg/g) and remained at a similar level following decellularization. In contrast, *L.S.* seaweed contained 3 mg/g of laminarin, which decreased to below 0.7 mg/g in the corresponding scaffold. The fucoidan content decreased by approximately 30% in the *L.D.* scaffold (from 13 mg/g in seaweed to 9 mg/g) whereas the *L.S.* scaffold showed a 166% increase relative to its seaweed (rising from 3 mg/g to 8 mg/g).

The mineral composition of the scaffolds shifted notably following decellularization (Table 3). The calcium (Ca) content increased substantially, with a 2.87-fold rise in *L.D.* (from 1.5% to 4.3% dry weight) and a 5-fold increase in *L.S.* (from 0.88% to 4.4%). In contrast, magnesium (Mg) levels declined moderately, from 0.74% to 0.27% in *L.D.* and from 0.50% to 0.24% in *L.S*. Sodium (Na), potassium (K), and iodine (I) concentrations were markedly reduced in both scaffold types, falling to below 0.1% dry weight (Table 3).

### 3.2. Biological Seaweed Scaffolds Retain Their Microstructural Features with Increased Porosity

**Figure 6:**
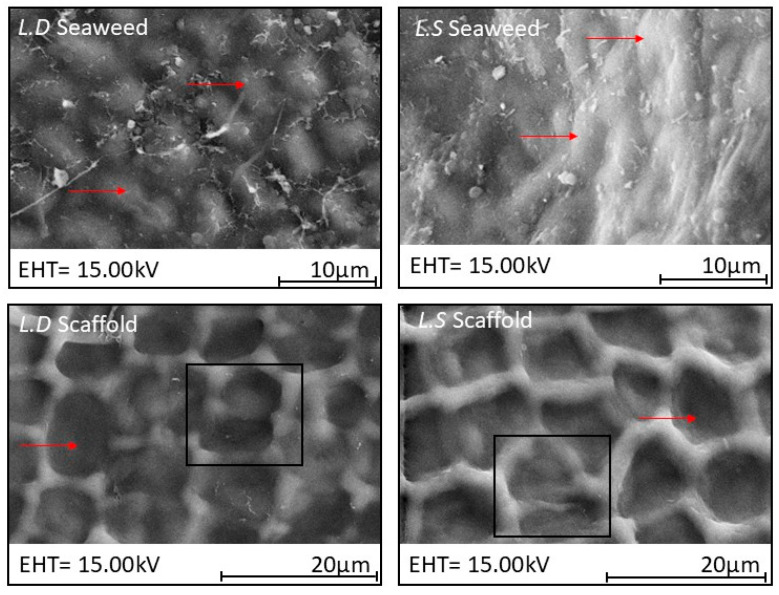
SEM images of the planar view of the *L.D*. and *L.S*. seaweed (**top panel**) and corresponding decellularized scaffolds (**bottom**). Red arrows, superimposed on the panels, showing the protruding surface chambers in the seaweed in comparison scaffold images (**bottom panel**), showing apparently collapsed mid-surface area features post decellularization. Black boxes in the scaffold images highlight partitioning within chamber, revealing compartmentalization of the scaffold structure following decellularization.

**Figure 7:**
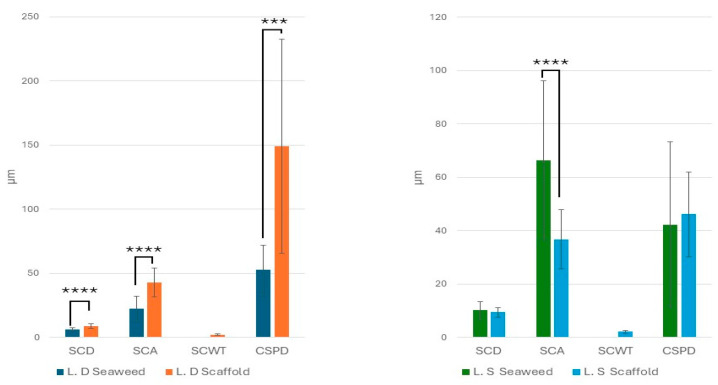
Mean values of Surface Chamber Diameter (SCD), Surface Chamber Area (SCA), and Cross-Sectional Pore Diameter (CSPD) are presented for *L.D.* and *L.S.* seaweeds in comparison to their respective scaffolds. Surface Chamber Wall Thickness (SCWT) values are presented only for *L.D.* and *L.S.* scaffolds. In the *L.D.* group, chamber dimensions (SCD, SCA, CSPD) significantly increased following decellularization, indicating structural expansion. Conversely, *L.S.* chambers remained largely unchanged post decellularization, with the exception of SCA, which exhibited a significant reduction. No statistical difference was noted between *L.D.* and *L.S.* SCWT. Statistical significance: *** *p* < 0.001, and **** *p* < 0.0001.

The microstructure of a biological scaffold is important to determine appropriate clinical application and best use within tissue engineering. The H&E staining was supported with comparative SEM and the micro-CT image analysis of the seaweed and scaffolds in an effort to gauge structural changes induced by the decellularization process. Microstructural analysis revealed a hierarchical chambered structure, characterized by well-defined internal compartments bounded by cell wall structures in both seaweed and scaffolds. In many regions of the scaffolds, smaller sub-chambers were observed nested within larger primary chambers, indicating a multilayered and spatially organized framework (Figure 6). Micro-CT images of the two scaffold types further revealed interconnected tunnels beneath the scaffold surface (Figure 7).

Welch’s ANOVA identified a significant difference in SCD across experimental groups (*p* = 6.78 × 10^−20^). Subsequent Games–Howell post hoc comparisons revealed that SCD was significantly lower in the *L.D.* seaweed compared to its corresponding scaffold (*p* = 3.71 × 10^−10^). In contrast, no statistically significant difference in SCD was observed between the *L.S.* seaweed and its scaffold (*p* = 0.861). Similarly, Welch’s ANOVA showed a significant variation in SCA among groups (*p* = 3.58 × 10^−30^). Post hoc analysis indicated a significant increase in SCA following decellularization in both the *L.D.* (*p* = 4.18 × 10^−10^) and *L.S.* (*p* = 2.72 × 10^−10^) scaffolds compared to their original seaweed, confirming that the decellularization process had a substantial impact on this parameter. For CSPD, Welch’s ANOVA also indicated a significant effect across groups (*p* = 4.75 × 10^−5^). Games–Howell post hoc testing revealed a significant increase in CSPD from the *L.D.* seaweed to its scaffold (*p* = 2.99 × 10^−4^) whereas no significant difference was detected between *L.S.* seaweed and its scaffold (*p* = 0.957), suggesting a scaffold-specific impact of the decellularization process. Finally, one-way ANOVA revealed no statistically significant difference in SCWT between *L.S.* and *L.D.* scaffolds (*p* = 0.705), indicating comparable wall thicknesses. Quantitative measurements of the Surface Chamber Diameter (SCD), Surface Chamber Area (SCA), Cross-Sectional Pore Diameter (CSPD), and Surface Chamber Wall Thickness (SCWT) are shown in Figure 7. The weight averages of the *L.D.* and *L.S.* scaffolds’ pore sizes, calculated from the SCD and CSPD measurements, were 48.9 ± 77.51 and 19.95 ± 17.72, respectively.

**Figure 8:**
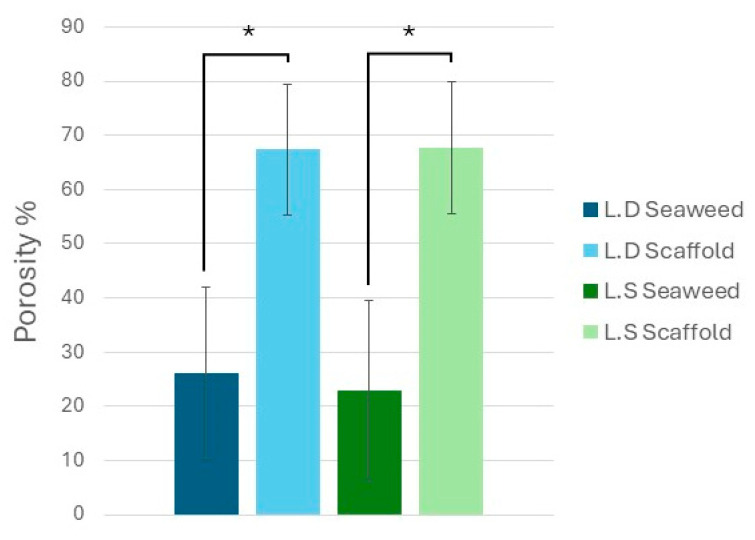
Porosity (%) of seaweed and scaffold samples derived from *Laminaria digitata* (*L.D*.) and *Laminaria saccharina* (*L.S*.). Bar graphs represent means ± standard deviations. A significant increase in porosity was observed following decellularization in both species, with *L.D.* and *L.S.* scaffolds showing significantly higher porosity than their respective seaweed controls (* *p* < 0.05). No significant difference was detected between *L.D.* and *L.S.* scaffolds.

Porosity testing of seaweed and scaffold samples following decellularization revealed a statistically significant difference among groups, as determined by one-way ANOVA. Tukey’s HSD post hoc analysis showed that the *L.S.* scaffold exhibited significantly higher porosity than its seaweed counterpart (*p* = 0.0210). Similarly, the *L.D.* scaffold had significantly greater porosity than *L.D.* seaweed (*p* = 0.0216), Figure 8. No significant difference in porosity was observed between the *L.S.* and *L.D.* scaffolds (*p* = 0.9999), nor between the seaweed species (*p* = 0.9926). These results confirm that the decellularization process significantly increased porosity in both seaweed species, and porosity remained statistically comparable post decellularization.

**Figure 9:**
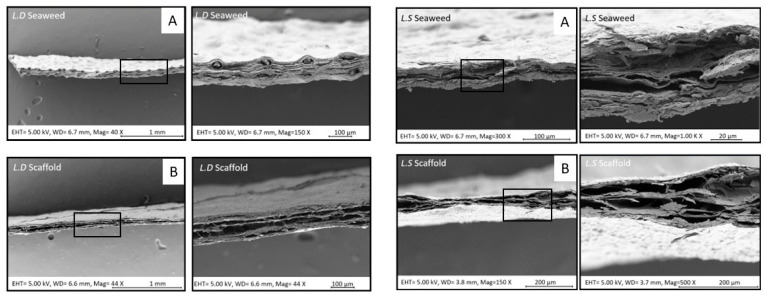
SEM cross-sectional comparison of the *L.D.* and *L.S.* seaweed sample (**A**, **top**) and the scaffold (**B**, **bottom**). A higher magnification of the boxed area is provided on the right. The images show the compact structure of the seaweed in comparison with the scaffold, emphasizing the increase in porosity of the scaffold.

**Figure 10:**
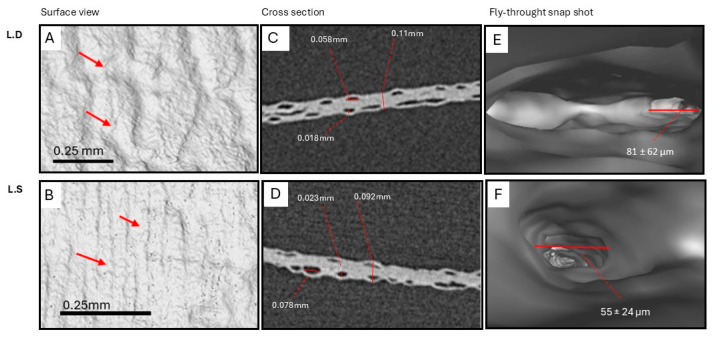
Micro-CT images illustrating interconnected tunnels within *L.D.* (**A**,**C**,**E**) and *L.S.* (**B**,**D**,**F**) scaffolds. (**A**,**B**) Surface views of the scaffolds with red arrows indicating the underlying tunnel structures. (**C**,**D**) Representative cross-sectional views showing measurements of tunnel diameter directly under the surface of the scaffold and the scaffold thickness. (**E**,**F**) Internal microstructure (fly-through views) with red lines superimposed across surface-adjacent tunnels to indicate average tunnel diameter.

Scanning electron microscopy (SEM) images (Figure 9) revealed clear differences in material compactness between the seaweed and the corresponding scaffolds. The seaweed exhibited dense, tightly packed microstructures whereas the scaffolds displayed a visibly more porous architecture. These structural differences underscore the impact of the decellularization process on the extracellular matrix organization and material properties. Notably, the effect of decellularization varied between *L.D.* and *L.S.*, suggesting species-specific responses in microstructural alteration. Complementing these observations, micro-computed tomography (micro-CT) images (Figure 10) further supported the SEM findings by demonstrating the internal scaffold morphology. The micro-CT scans revealed interconnected tunnel-like voids beneath the scaffold surfaces, consistent with enhanced internal porosity and potential for improved fluid transport and cell infiltration.

In the *L.D.* scaffold, the tunnel diameter directly underneath the seaweed surface averaged 81 ± 62 µm (n = 28), while in the *L.S.* scaffolds, diameters averaged 55 ± 24 µm (n = 22) (Figure 10). No significant difference was found between the tunnel diameter of the *L.D.* and *L.S.* scaffolds (*p* = 0.0684), although a trend toward larger tunnel sizes in the *L.D.* scaffolds was observed.

### 3.3. The L.D. and L.S. Biological Scaffolds Have Similar Swelling Properties but Different Fluid Absorption Properties

**Figure 11:**
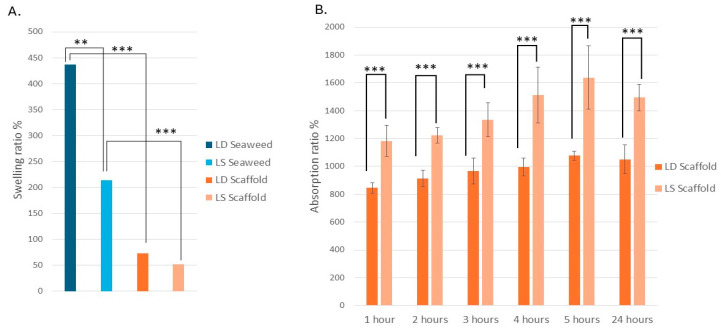
(**A**) Swelling ratio (%) after 2 h rehydration in 0.9% NaCl saline, indicating significant differences between the seaweed and scaffold groups and between the *L.D.* and *L.S.* seaweed. No significant difference was observed between the swelling ratios of scaffold groups. (**B**) Absorption ratio of scaffolds over 24 h, showing more than 10-fold increase in weight. The *L.S.* scaffold demonstrated the highest absorption capacity. Statistical significance: ** *p* < 0.01, *** *p* < 0.001.

To assess the impact of decellularization on swelling behavior, sample thickness was recorded and swelling ratios were determined following 2 h of rehydration in 0.9% (*w*/*v*) NaCl solution. This procedure was applied to both the seaweed samples (*L.D.* and *L.S.*) and their respective scaffolds. Welch’s ANOVA revealed a statistically significant difference in swelling ratios among the groups (*p* = 2.4 × 10^−6^). Post hoc comparisons using the Games–Howell test showed that *L.D.* seaweed exhibited the highest swelling ratio, with an increase in thickness of approximately 450% (from 0.16 ± 0.08 mm to 0.88 ± 0.12 mm), which was significantly greater than its corresponding scaffold (0.17 ± 0.05 mm to 0.30 ± 0.02 mm) (*p* = 0.0003) and all other groups. *L.S.* seaweed exhibited a moderate swelling ratio of approximately 208% (from 0.12 ± 0.02 mm to 0.37 ± 0.12 mm), with significantly greater swelling compared to its corresponding scaffold (from 0.08 ± 0.02 mm to 0.13 ± 0.03 mm; *p* = 0.0001) (Figure 11A). No significant difference was however noted between the swelling ratios of the two scaffold types (*p* = 0.582), indicating similar fluid uptake behavior post decellularization.

Further testing on the scaffolds showed distinct fluid absorption performances when immersed in 0.9% NaCl over 24 h. A repeated-measures ANOVA showed significant main effects of scaffold type (*p* = 3.57 × 10^−4^) and time (*p* < 0.001) on weight gain (absorption ratio), along with a significant scaffold × time interaction (*p* < 0.001). This indicates that overall fluid uptake differed between the two scaffold compositions and changed significantly with immersion duration. In other words, *L.S.* and *L.D.* scaffolds exhibited different absorption trends over time. These findings were further supported by a linear mixed-effects model analysis. The mixed-effects model confirmed significant main effects of group (F(1, 6) = 52.18, *p* < 0.001) and time (F(6, 36) = 226.59, *p* < 0.001), as well as a significant group × time interaction (F(6, 36) = 8.97, *p* < 0.001). Together, the ANOVA and mixed-model results demonstrate that both scaffold composition and immersion duration independently influence fluid uptake and that *L.S.* and *L.D.* scaffolds respond differently over the immersion period. Post hoc comparisons clarified the nature of these differences over time. At the initial measurement (t0, baseline), there was no significant difference in absorption between *L.S.* and *L.D.* (both essentially dry). However, at every subsequent time point (1–5 h and 24 h), *L.S.* scaffolds absorbed significantly more fluid than *L.D.* scaffolds (*p* < 0.001 for each time point beyond t0). By the end of the 24 h period, *L.S.* scaffolds reached an average of 1494.3% weight gain (relative to their dry weight) compared to 1050.7% for *L.D.* scaffolds. This finding underscores the substantially greater fluid uptake capacity of the *L.S.* material. The absorption kinetics differed primarily in the early phase of immersion. Both scaffold types exhibited the most rapid weight gain during the first hour of soaking, indicating an initial burst of fluid uptake. Thereafter, the rate of increase in absorption slowed considerably. Notably, no significant differences were detected between adjacent hourly time points during the 0–5 h period, suggesting that the scaffolds’ hydration began to plateau after the initial hour. By the later hours (5 h through 24 h), weight gain approached a steady state with only minimal further increases. Figure 11B illustrates the time-course absorption profiles of *L.S.* and *L.D.* scaffolds. *L.S.* consistently maintained higher absorption values over time, and both curves flattened as they approached the 24 h mark.

The decellularization process led to a marked reduction in swelling capacity for both seaweed species, with a more pronounced effect observed in the *L.D.* samples. These results underscore the differential responses of *L.D.* and *L.S.* seaweed to processing, which may affect their functional properties as scaffolds. While *L.D.* seaweed exhibited the highest swelling ratio prior to decellularization, both resulting scaffolds displayed significantly reduced and comparable swelling behavior. In terms of fluid absorption, the *L.S.* scaffold outperformed the *L.D.* scaffold over 24 h, with both showing the greatest fluid uptake during the first hour of immersion, followed by a plateauing trend.

### 3.4. Strength and Stiffness Increased in Both Scaffolds, with Strain Enhancement Observed in L.S.

**Figure 12:**
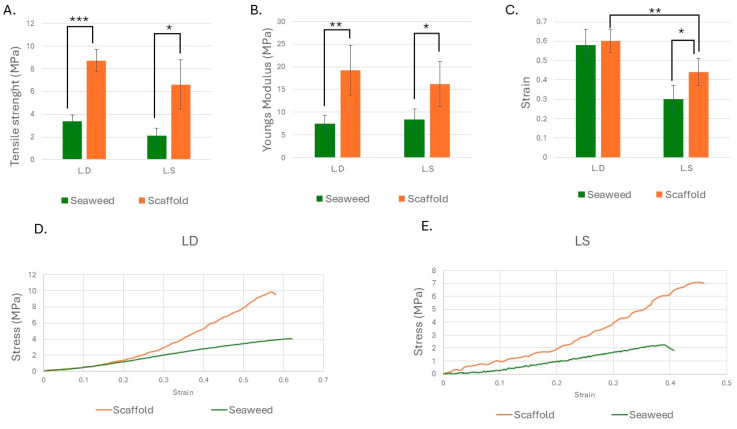
Mechanical characterization of seaweed and scaffold samples. (**A**) Tensile strength and (**B**) Young’s modulus were significantly increased in both *L.D.* and *L.S.* scaffolds compared to their respective seaweeds. (**C**) Strain analysis revealed preserved ductility in the *L.D.* scaffold while the *L.S.* scaffold demonstrated a significant increase in extensibility relative to its seaweed and the *L.D.* scaffold. (**D**,**E**) show representative stress–strain curves for *L.D.* and *L.S.* groups, respectively, illustrating distinct mechanical behavior pre- and post-decellularization conditions. n = 6 per group. Statistical significance: * *p* < 0.05, ** *p* < 0.01, and *** *p* < 0.001.

Six specimens from each of the four groups: seaweed, L.D. scaffold, L.S. seaweed, and L.S. scaffold, were subjected to uniaxial tensile testing. Stress-strain curves were produced to evaluate the ultimate tensile strength and the elastic modulus (Young’s modulus) for each group. Welch’s ANOVA revealed statistically significant differences in both ultimate tensile strength (*p* = 7.04 × 10^−7^) and elastic modulus (*p* = 0.001) among the groups. The *L.D.* scaffold exhibited a greater mechanical performance enhancement compared to its seaweed than the *L.S.* scaffold, with increases of approximately 156% in the elastic modulus (*p* < 0.01) and 158% in tensile strength (*p* < 0.001). In comparison, the *L.S.* scaffold showed significant improvements of 92% in the elastic modulus and 214% in tensile strength (both *p* < 0.05) relative to its corresponding seaweed. No significant difference in tensile strength or stiffness was observed between the *L.D.* and *L.S.* scaffolds. These comparisons are shown in Figure 12A,B.

Strain measurements revealed statistically significant variation across groups, as confirmed by one-way ANOVA (*p* = 7.44 × 10^−7^). The *L.D.* scaffold retained strain capacity similar to that of its original seaweed (*p* = 0.952), indicating preserved ductility following decellularization. In contrast, the *L.S.* scaffold exhibited a 47% increase in strain compared to its original seaweed (*p* = 0.01) and significantly higher strain than the *L.D.* scaffold (*p* = 0.004), suggesting enhanced extensibility specific to the *L.S.* scaffold. These differences in strain and corresponding stress vs. strain responses are illustrated in Figure 12C–E.

## 4. Discussion

### 4.1. Sequence and Species-Dependent Visible-Light Decellularization

Visible-light decellularization efficiently eliminated cellular components while largely preserving both the micro- and macrostructure in *Laminaria digitata* and *Laminaria saccharina*. Histological analysis confirmed that the resulting acellular scaffolds retained an intact extracellular matrix, thereby reducing immunogenicity for applications as biomaterials [10,23,33]. Across nine light regimens, efficacy was sequence- and species-dependent: *L.D*. cleared most effectively with red → blue (R→B) or (R + B)→R whereas *L.S.* most effective light regiment was R→(R + B) for the joint removal of cells and debris (Figure 4 and Figure 5). Histological analysis revealed that control seaweed samples, which were not exposed to light but were submerged in turbulent water for four weeks, retained cells embedded within the extracellular matrix (ECM). The removal of cells from seaweed tissue is likely attributed to the combined effects of light exposure and continuous turbulent water rinsing. A plausible explanation of the effect of the lights involves wavelength-dependent photochemical processes and variations in penetration depth. Red light (620 nm) is capable of penetrating further into turbid tissues and has been observed to induce intracellular photo-oxidative damage [44]. Subsequent blue or combined red-and-blue light (470 nm; R + B) might induce the generation of reactive oxygen species (ROS) causing cell death as has been shown in previous studies [45]. Over a period of four weeks, the two lights would induce cell rupture and facilitate the removal of cell debris from the extracellular matrix (ECM). This hypothesis will need to be supported by further research.

### 4.2. Choosing the Default Decellularization Method of Scaffold Production

The method R→B (620 nm→470 nm; 2 weeks each) was selected as the default preparation to facilitate cross-species comparability and streamline production. The sequence demonstrated robust scaffold clearance in both species with preserved ECMs. Notably, *L.S.* showed its best combined cell-and-debris clearance with R→(R + B), indicating that the species-specific method may further reduce residual debris or processing times. Cell clearance from scaffolds helped identify strong candidates, but thorough evaluation will require further analysis, such as by measuring residual DNA and double-stranded DNA.

### 4.3. Scaffold Macro- and Microstructure Preservation and Implications for Recellularization

H&E and SEM analysis confirmed that the scaffolds microstructures remained largely intact following decellularization while exhibiting increased porosity compared to the original seaweed. The retention of microstructural integrity, combined with the efficient removal of cellular components and minimal detergent-associated matrix loss or residue, indicates that seaweed scaffolds may support host cell infiltration and demonstrate biocompatibility characteristics desirable for regenerative medicine applications [11,26,39]. Compared with standard SDS/Triton plant decellularization, a light-only process removes the risk of residual surfactants and may simplify scaffold production and biocompatibility testing [46].

### 4.4. The Scaffolds’ Selective Retention and/or Depletion of Polysaccharides

The effect of decellularization on the chemical composition of the seaweed scaffold showed a relative decrease in some components and an increase in others. *L.S.* scaffolds demonstrated a moderate relative increase in total protein content while *L.D.* scaffolds largely retained their original protein levels.

The polysaccharide composition underwent marked alterations during the decellularization process, as summarized in Table 2. Notably, fucoidan content decreased by approximately 30% in the *L.D.* scaffold (from 13 mg/g to 9 mg/g), whereas the *L.S.* scaffold exhibited a 166% increase compared to its seaweed (rising from 3 mg/g to 8 mg/g). This preservation and enrichment in the case of *L.S.* suggest that fucoidan is not uniformly depleted by decellularization and may be retained selectively depending on species-specific matrix properties. Due to fucoidans’ bioactive nature (e.g., angiogenic/osteogenic modulation depending on chain length and sulfation), their retention may be advantageous, and this merits the targeted characterization of molecular weight and sulfate content [29,30,31].

Alginate content also decreased across both scaffold types, with a 25% reduction in *L.D.* (from 85 mg/g to 64 mg/g) and a 48% reduction in *L.S.* (from 63 mg/g to 33 mg/g). This data suggests that approximately ~50–75% of the original alginate content remains embedded in the scaffold structure. The difference in alginate reduction likely reflects species-specific cell wall structure and relative concentration after the leaching of soluble fractions.

Other polysaccharides including mannitol, laminarin, cellulose, and galactose demonstrated species-dependent shifts, with mannitol and laminarin showing significant depletion for both scaffold types, which was expected given their solubility and cytoplasmic localization. Cellulose showed a relative increase in the *L.S.* scaffold (26% to 44% dry weight) while decreasing modestly in *L.D.* (34% to 31% dry weight). These results suggest that the decellularization preferentially removes cytoplasmic and non-structural scaffold components while leaving behind the cell-wall framework rich in cellulose, alginate, and proteins (Table 2).

### 4.5. Mineral Shifts and Alginate–Calcium Interactions in the Scaffolds

After decellularization, there was a pronounced decline in the mineral content of both scaffold types. Iodine was nearly completely removed by the decellularization process (*L.S.*: 0.25% to 0.04%) in the scaffold, with a similar reduction observed for *L.D.* (0.12% to 0.04%). Potassium and sodium levels were substantially reduced in the scaffolds compared to their respective seaweeds following decellularization. In *L.D.* and *L.S.* scaffolds, K and Na dropped by more than half relative to the seaweed samples (*L.D.*; K: 3% to 0.025% and Na: 1.6% to 0.097%). Such losses are to be expected due to the monovalent ions that are highly soluble and readily diffuse out of the material during the decellularization process. Indeed, significant ash (minerals) reductions (~40–50% loss) observed during processing of kelp in other studies have been attributed to the leaching of iodine and salts like Na and K [47]. In contrast to other minerals, magnesium exhibited a smaller relative decrease, while calcium content showed a marked relative increase following decellularization. For the *L.D.* samples, decellularization led to an increase in calcium content from 1.5% in the seaweed to 4.3% in the scaffold, while magnesium levels decreased from 0.74% in the seaweed to 0.27% in the scaffold. Similarly, in the *L.S.* samples, calcium content rose from 0.88% in the seaweed to 4.4% in the scaffold, and magnesium decreased from 0.50% in the seaweed to 0.24% in the scaffold. This relative enrichment of calcium, and the partial retention of magnesium, is likely due to their stronger binding to the alginate-rich cell wall matrix. Divalent cations such as Ca^2+^ form stable ionic cross-links with guluronate blocks in alginate, making them less susceptible to removal during decellularization [48]. Thus, the apparent increase in calcium reflects a relative concentration effect due to the loss of more soluble components, rather than an absolute gain (Table 3). Functionally, retained Ca in an alginate-rich scaffold may have beneficial hemostatic use in clinical setting and could support osteoconductive templating [49].

### 4.6. Scaffolds’ Microstructural Features and Porosity

Quantitative image analysis (H&E/SEM/micro-CT) indicated that visible-light decellularization preserves the hierarchical and chambered structure of *L.D.* and *L.S.* while increasing overall porosity. Hierarchy is evident as smaller sub-chambers are nested within larger surface chambers (Figure 6), with interconnected tunnels immediately beneath the surface (Figure 10). Statistically, Welch’s ANOVA showed highly significant group effects for SCD and SCA (*p* = 6.78 × 10^−20^ and 3.58 × 10^−30^), with *L.D.* scaffolds exhibiting larger SCD, SCA, and CSPD values than their seaweeds (all *p* < 0.001), consistent with structural expansion following decellularization. In contrast, *L.S.* scaffolds showed a selective decrease in SCA (*p* = 2.72 × 10^−10^) while SCD and CSPD were unchanged, indicating a species-specific remodeling of surface chamber geometry. Porosity increased significantly in both species after decellularization (Tukey’s HSD *p* ≈ 0.02 for each), with no difference between the two scaffold types (*p* = 0.9999). The SCWT was comparable between *L.D.* and *L.S.* scaffolds (*p* = 0.705) (Figure 7). Micro-CT identified a network of interconnected internal tunnels (*L.D.*: 81 ± 62 µm; *L.S*: 55 ± 24 µm). However, SEM analysis showed larger Cross-Sectional Pore Diameter (CSPD) values (*L.D.*: 149.25 ± 83.50 µm; *L.S.*: 46.18 ± 15.83 µm). Notably, the tunnel dimensions close to the surface of the scaffold observed by Micro-CT fell within the broader standard deviation range of the SEM-derived CSPD measurements. While the two modalities captured distinct aspects of the scaffold microarchitecture, this overlap suggests a general consistency in structural characterization. Method-comparison studies consistently report such modality-dependent shifts and recommend reporting both distributions and connectivity metrics for porous scaffolds [50].

### 4.7. Species -Specific Decellularization Effect on Microstructure: Proposed Mechanistic Insights

The divergent microstructural responses (expansion in *L.D.*; selective SCA reduction in *L.S.*) are plausibly governed by wall chemistry and cross-linking that differ by to two seaweed species. As demonstrated in previous sections, *L.S.* exhibited higher cellulose enrichment and greater Ca^2+^ increases than *L.D.* following decellularization. These conditions are anticipated to enhance the rigidity of alginate–cellulose networks through Ca^2+^ “egg-box” junctions and diminish wall compliance, which aligns with the observed restricted geometric expansion in *L.S.* [48]. By comparison, the modest elevation in Ca^2+^ levels and the moderate decrease in cellulose measured in *L.D.* corresponded to a cell wall exhibiting greater compliance. This compliance could facilitate the dilation of surface chambers and internal pores during the decellularization process, resulting in substantial increase in SCD, SCA, and CSPD values. Although direct evidence would require further mechanical testing and ion-exchange analysis, the observed correlation between geometry and the chemical profile of the scaffolds aligns with established alginate–Ca^2+^ interactions previously observed.

### 4.8. Functional Implications of the Scaffold’s Microstructure and Clinical Application

The scaffolds exhibited multiscale porosity, comprising micro-pores near the surface (~10 µm), subsurface tunnels of 50–100 µm, and an extended distribution up to roughly 150 µm. In porous materials, small pores support protein adsorption and initial cell attachment while larger pores (~100–300 µm) promote cellular migration, angiogenesis, and bone integration [51,52]. However, the maximum pore size in *L.D.* scaffolds (~149 µm) falls below the ideal 300–400 µm range associated with optimal bone ingrowth [53].

### 4.9. Comparisons to Plant-Derived and Clinical Scaffolds

The pore size ranges observed in our scaffolds (8.8 ± 1.8 to 162.2 ± 89.3 µm) were broadly comparable to those of other plant-derived scaffolds, such as decellularized carrots, celery, and apples (70 ± 12 to 420 ± 33 µm) [54,55], and to biological ECM-based scaffolds like bovine tendon collagen (≈132 µm) [56]. In contrast, synthetic scaffolds such as polyurethane-based BTM (589 ± 297 µm) and small intestinal submucosa (SIS) sheets (379.2 ± 34.8 µm) [56,57] have exhibited substantially larger pore sizes (Table 4), highlighting the diversity of pore microstructure currently used within tissue TE and RM applications.

### 4.10. Scaffold Swelling and Absorption Performance

The swelling ratio demonstrated that seaweed samples exhibited greater expansion in thickness upon rehydration compared to the scaffolds; however, no significant difference was observed between the two scaffold types. This discrepancy is likely attributable to the differing chemical compositions of the seaweed and scaffold materials. Specifically, the seaweed contained higher concentrations of hydrophilic polysaccharides (such as alginate and mannitol) than the scaffolds, which may explain the increased thickness observed in rehydrated seaweed relative to the scaffolds [58].

### 4.11. Time-Dependent Kinetics: Early Burst Then Plateau

The scaffolds’ absorption characteristics were of specific interest due to potential clinical application leading to the additional testing of the scaffolds’ absorption properties over 24 h. Notably, *L.S.* scaffolds absorbed 1494% of their dry weight, surpassing 1050% seen in the *L.D.* scaffolds, with an early 0–1 h uptake burst followed by a plateau (Figure 11). The rapid 0–1 h mass gain followed by a gradual approach to a steady state was consistent with Lucas–Washburn-type capillary imbibition, where penetration scales with √t until viscous and capillary forces balance or pores saturate; related medical-foam studies have reported similar semi-quantitative agreement with Washburn kinetics [59]. The subsequent plateau reflects near-equilibrium between capillary forces, viscosity, and any slow counter-ion exchange.

### 4.12. Comparison with Related Scaffolds and Dressings

Plant-derived scaffolds span a wide range of water-uptake behaviors. Recent work on decellularized baby spinach leaves has reported ~1696% water uptake within 120 min (DI water, 37 °C) [60], similar to the 24 h saline absorption observed here, acknowledging that saline depresses swelling relative to DI water. Clinically, alginate dressings are valued for high exudate capacity, often absorbing 15–20× their weight, a performance linked to their porous structure and Ca^2+^/Na^+^ exchange at the wound surface; the scaffold absorption ratios reported here fall into the same functional class [61]. *L.S.* scaffolds, in particular, show greater sustained uptake, and may suit moderate-to-high exudate scenarios, whereas *L.D.* scaffolds provide more restrained fluid loading. These distinctions may also matter for drug-loading/release from hydrated matrices [58,62,63].

### 4.13. Clinical Indications Suggested by the Swelling and Absorption Data

For clinical applications, the absorption properties of wound dressings are important to maintain appropriate moisture levels in wounds. Correct moisture in wounds will accelerate healing, diminish discomfort, and enhance cosmetic outcomes [64].

### 4.14. The Mechanical Properties of the Seaweed Scaffolds

The *L.D.* and *L.S.* scaffolds exhibited J-shaped stress–strain curves (Figure 12), characteristic of soft biological tissues such as arteries, skin, and tendons [65,66,67]. This curve morphology reflected an initially compliant phase, where the material deformed readily under low stress, followed by progressive stiffening with increasing load. This pattern was accentuated following decellularization.

Both scaffold types demonstrated significantly improved mechanical performance compared to their original seaweeds. Specifically, *L.D.* scaffolds showed a 156% increase in Young’s modulus (*p* < 0.01) and a 158% increase in tensile strength (*p* < 0.001), while *L.S.* scaffolds exhibited a 92% increase in the elastic modulus and a 214% increase in tensile strength (*p* < 0.05).

The observed increase in the elastic modulus and strength following decellularization may be attributed to several factors identified in prior sections. Firstly, the elimination of cellular components and subsequent matrix compaction likely enhance the density of the load-bearing network. Secondly, compositional analysis reveals that the scaffold retains both cellulose and alginate and exhibits elevated Ca^2+^ content compared to native tissue (Table 3). Increased Ca^2+^ would raise the fraction of alginate “egg-box” junctions, which is expected to increase stiffness and reduce low-strain compliance, yielding higher stress at a given strain and a steeper initial slope for the stress–strain curve. In addition, visible-light exposure during decellularization may promote mild oxidative cross-linking in wall components, further contributing to the observed mechanical gains [68]. Additionally, the loss of cytoplasmic solutes (e.g., mannitol/laminarin) (Table 2) may reduce matrix plasticization, and increase stiffness and strength, consistent with the results (Figure 12). Furthermore, the partition chambers and tunnels that are part of the seaweed’s microstructure can act as micro-domains that sequentially engage under strain. Species-specific geometry and the biochemical profile of the scaffolds are likely to determine when and how these domains are recruited under stress.

The strain at break differed between the two scaffold types. The *L.D.* scaffold maintained strain-at-break values comparable to its seaweed (0.58 vs. 0.60, *p* = 0.952), suggesting that ductility was preserved despite increased stiffness. In contrast, *L.S.* scaffolds demonstrated a significant 47% increase in strain (0.44 vs. 0.30, *p* = 0.01) and higher strain at failure compared to *L.D.* (*p* = 0.004), highlighting their potential to combine mechanical strength with flexibility. These differences likely reflect the different chemical profiles of the scaffolds and seaweeds and possible cross-linking during decellularization.

### 4.15. Mechanical Properties and Clinical Relevance

Mechanical characteristics are especially relevant for scaffolds that are intended to replicate the behavior of load bearing and viscoelastic tissues that are subjected to cyclic deformation, such as skin, vasculature, and fascia. From a clinical standpoint, scaffold design should reflect the mechanical demands of the target tissue as much as possible. For example, human skin exhibits tensile strength ranging from 6.4–21.6 MPa [69] while arteries typically range between 1.44 MPa and 3.08 MPa [70] depending on individual health and age. Given this variability, scaffold mechanical properties must be application-specific. Wound dressings prioritize moisture retention and bacterial exclusion over tensile strength while scaffolds for hernia repair or ligament replacement require significantly higher mechanical integrity. In this context, the tensile strength values achieved by seaweed scaffolds fall within the clinically relevant range for soft tissue repair. Compared to commercially available wound-healing grafts, seaweed-derived scaffolds offer superior stiffness and competitive strength. After 2 h of hydration, *L.D.* and *L.S.* scaffolds exhibited Young’s modulus values of 19.19 ± 5.51 MPa and 16.19 ± 4.94 MPa, respectively, higher than the reported values for wet bovine (0.012 ± 0.003 MPa). Fish skin grafts (10.1 ± 1.8 MPa) [71] and decellularized human dermis (AlloDerm^®^, 14.3 MPa) [72] have exhibited higher tensile strength; the *L.D.* scaffold achieved 8.73 ± 0.98 MPa, approaching the range observed for fish skin and falling within a clinically relevant spectrum for soft tissue repair (Table 5). As rehydration conditions for the commercial biological scaffolds are unreported, direct comparisons should be interpreted with caution, though they remain contextually informative.

The *L.D.* scaffold offers a combination of strength, stiffness, and preserved ductility, making it suitable for applications requiring mechanical compliance without compromising structural integrity. In contrast, the *L.S.* scaffold achieves simultaneous increases in strength and extensibility, potentially benefiting applications where both resilience and flexibility are required.

## 5. Conclusions

This study showed that visible-light decellularization produces acellular *Laminaria digitata* and *Laminaria saccharina* scaffolds with intact microstructure and increased porosity. Both scaffold types showed stress–strain responses characteristic of soft tissues, with significant increases in tensile strength and Young’s modulus, suggesting clinical potential for soft tissue repair. The results are summarized in the following:

- *L.D.* offered increased stiffness and strength without losing ductility; *L.S.* achieved more strength along with enhanced extensibility.
- Histological, SEM, and micro-CT analyses verified that the original layered microstructure and internal tunnel networks were preserved.
- Biochemical analysis showed reduced mannitol, laminarin, and sodium in seaweed, alongside a relative increase in calcium, likely due to its strong binding to the alginate matrix.
- Fucoidan content was maintained or even relatively increased in *L.S.* scaffolds, potentially supporting future bioactivity.
- Both scaffolds were highly hydrophilic: *L.S.* absorbed 1494% fluid in 24 h while *L.D.* showed greater swelling, a key property for wound healing and exudate management.

Regarding practical application, the results indicate that the *L.S.* scaffolds are well-suited for wound care and skin repair due to their strong exudate absorption and conformability while *L.D.* scaffolds are recommended for reinforcing soft tissues, particularly when greater stability and stiffness are needed. Interconnected tunnels may allow early bone and blood vessel growth, but larger pores are likely needed for significant bone ingrowth.

The brown seaweeds *L.D.* and *L.S.* are widely accessible for sustainable harvesting or aquaculture. The decellularization process employs environmentally responsible resources, specifically visible light and water without the use of chemicals, thereby supporting scalability and industrial feasibility. The next essential translation steps will involve reducing production time, establishing batch-to-batch quality control (including DNA/endotoxin testing and mechanical evaluation), and confirming sterilization methods that are compatible with alginate–cellulose matrices. Additionally, future in vitro and in vivo studies will evaluate the biocompatibility, efficacy, clinical application, and usability of the scaffolds.

## Figures and Tables

**Table 1:** Visual electromagnetic radiation sequence exposure outlined.

Light Sequence	Description
blue and red → blue	Combined blue and red light for two weeks, followed by blue light for two weeks
blue	Blue light exposure for four weeks
blue → blue and red	Blue light for two weeks, followed by combined blue and red light for two weeks
blue → red	Blue light for two weeks, followed by with red light for two weeks
red → blue and red	Red light for two weeks, followed by combined blue and red light for two weeks
blue and red	Red and blue light exposure for four weeks
red	Red light exposure for four weeks
red → blue	Red light exposure for two weeks, followed by blue light for two weeks
blue and red → red	Combined blue and red light for two weeks, followed by red light for two weeks

**Table 2:** Comparison of biochemical composition between seaweed and decellularized scaffolds derived from *Laminaria digitata* (*L.D*.) and *Laminaria saccharina* (*L.S*.), including protein content (% of total dry weight: dw), carbohydrate components (mannitol, fucoidan, alginate, laminarin, galactose), and cellulose.

Sample	Type	Protein (% dw)	Mannitol (mg/g)	Fucoidan (mg/g)	Alginate (mg/g)	Cellulose (% w/w)	Laminarin (mg/g)	Gal (mg/g)
*L.D.*	seaweed	5.4	0.67	13	85	34	<0.7	0.028
	scaffold	5.1	0.24	9	64	31	<0.7	0.032
*L.S.*	seaweed	3.6	25	3	63	26	3	0.85
	scaffold	5.5	0.25	8	33	44	<0.7	0.092

**Table 3:** Comparison of mineral composition (% of total dry weight) between seaweed and decellularized scaffolds from *Laminaria digitata* (*L.D*.) and *Laminaria saccharina* (*L.S*.).

Sample	Type	Ca (%)	Mg (%)	Na (%)	K (%)	I (%)
*L.D.*	seaweed	1.5	0.74	1.6	3.0	0.12
	scaffold	4.3	0.27	0.097	0.025	0.04
*L.S.*	seaweed	0.88	0.5	2.4	4.4	0.25
	scaffold	4.4	0.24	0.082	0.025	0.04

**Table 4:** Pore size comparison of biological scaffold materials. Values are reported as means ± standard deviations.

Biomaterial	Ave Pore Size (μm)
Brown seaweed *L.D.*	48.9 ± 77.5
Brown seaweed *L.S*.	19.95 ± 18.72
Green seaweed *Ulva* sp.	20.2 ± 4 [39]
Apples	420 ± 33 [54]
Celery	125 ± 11 [54]
Carrots	70 ± 12, 130 ± 26 [54]
Collagen–GAG, Integra^®^ Dermal Reg.	132 ± 91 [56]
Biodegradable Temporizing Matrix, BTM NovoSorb^®^	589 ± 297 [56]
Porcine Small Intestine Submucosa (SIS)	379.2 ± 34.8 [57]

**Table 5:** Mechanical properties of ECM and biological scaffolds rehydrated.

Biomaterial	σ_uts_ (MPa)	Young’s Modulus (MPa)	Strain at σ_uts_
Brown seaweed *L.D*	8.73 ± 0.98	19.19 ± 5.51	48.9 ± 77.5
Brown seaweed *L.S*	6.61 ± 2.16	16.19 ± 4.94	19.95 ± 18.72
AlloDerm^®^	14.3	NA	20.2 ± 4 [72]
Kerecis Fish skin	10.1 ± 1.8	0.67 ± 0.2	420 ± 33 [71]
Proheal Bovine collagen	0.047 ± 0.003	0.012 ± 0.003	125 ± 11 [71]

Human decellularized dermis.

## Data Availability

The raw data supporting the conclusions of this article will be made available by the authors on request.

## Abbreviations

The following abbreviations have been used in this manuscript:

*L.D*.	*Laminaria digitata*
*L.S*.	*Laminaria saccharina*
SCD	Surface Chamber Diameter
SCA	Surface Chamber Area
CSPD	Cross-Sectional Pore Diameter
SCWT	Surface Chamber Wall Thickness
SEM	Scanning electron microscopy
Micro-CT	Micro-computed tomography
H&E	Hematoxylin and eosin staining
W_0_	Initial weight
W_1_	Test weight
W_i_	Weight at time i
σ_uts_	Tensile strenght
E	Elastic modulus or Young’s modulus
ε	Strain
